# Supplementary material for: Polish Translation and Validation of the Voice Handicap Index (VHI-30)
Source: Int J Environ Res Public Health. 2022 Aug 29;19(17):10738. doi: 10.3390/ijerph191710738 (PMC9518103; doi:10.3390/ijerph191710738)
Supplement: Supplementary file 1 [file ijerph-19-10738-s001.zip › ijerph-1825726-supplementary.pdf]

Autorzy: B. H. Jacobson, A. Johnson, C. Grywalski, A. Silbergleit, G. Jacobson, M. S. Benninger, C. W. Newman

Indeks niepełnosprawności głosowej
Voice Handicap Index (VHI-POL)

…………………………………………………….   wiek ……. płeć: M / K   data:………….…….

Poniższe stwierdzenia używane są przez wielu ludzi do opisu swojego głosu oraz jego wpływu na ich życie. Prosimy o zakreślenie odpowiedzi wskazującej, jak często miał/a Pan/i podobne odczucia.

Poszczególne cyfry oznaczają:

0 – nigdy
1 – prawie nigdy
2 – czasami
3 – prawie zawsze
4 – zawsze

F1.	Z powodu mojego głosu innym ludziom jest trudno mnie usłyszeć	0	1	2	3	4
P2.	Kiedy mówię, brakuje mi powietrza	0	1	2	3	4
F3.	Ludzie mają problem ze zrozumieniem mnie w hałaśliwych pomieszczeniach	0	1	2	3	4
P4.	Brzmienie mojego głosu zmienia się w ciągu dnia	0	1	2	3	4
F5.	Moja rodzina ma problem z usłyszeniem mnie, gdy wołam do nich z innej części domu	0	1	2	3	4
F6.	Korzystam z telefonu rzadziej, niż bym chciał/a	0	1	2	3	4
E7.	Kiedy rozmawiam z innymi ludźmi, jestem spięty/a z powodu mojego głosu	0	1	2	3	4
F8.	Unikam zbiorowisk ludzi ze względu na mój głos	0	1	2	3	4
E9.	Mój głos wydaje się irytować innych	0	1	2	3	4
P10.	Ludzie pytają: “Czy coś jest nie tak z twoim głosem?”	0	1	2	3	4
F11.	Z powodu mojego głosu rzadziej rozmawiam z przyjaciółmi, sąsiadami czy rodziną	0	1	2	3	4
F12.	Ludzie proszą mnie o powtórzenie wypowiedzi podczas rozmowy twarzą w twarz	0	1	2	3	4

P13.	Mój głos jest skrzypiący i szorstki	0	1	2	3	4
P14.	Odczuwam wysiłek podczas tworzenia głosu	0	1	2	3	4
E15.	Sądzę, że inni ludzie nie rozumieją mojego problemu z głosem	0	1	2	3	4
F16.	Moje problemy z głosem ograniczają moje życie osobiste i towarzyskie	0	1	2	3	4
P17.	Czystość mojego głosu jest nie do przewidzenia	0	1	2	3	4
P18.	Staram się zmieniać (modulować) mój głos, żeby brzmiał inaczej	0	1	2	3	4
F19.	Czuję się wykluczony/a z rozmów z powodu mojego głosu	0	1	2	3	4
P20.	Mówienie jest dla mnie bardzo dużym wysiłkiem	0	1	2	3	4
P21.	Mój głos jest gorszy wieczorem	0	1	2	3	4
F22.	Z powodu mojego głosu tracę dochody	0	1	2	3	4
E23.	Moje problemy z głosem martwią mnie	0	1	2	3	4
E24.	Jestem mniej towarzyski/a z powodu moich problemów z głosem	0	1	2	3	4
E25.	Z powodu mojego głosu czuję się niepełnosprawny/a	0	1	2	3	4
P26.	Mój głos „odmawia mi posłuszeństwa” w trakcie mówienia	0	1	2	3	4
E27.	Drażni mnie, gdy ludzie proszą mnie o powtórzenie	0	1	2	3	4
E28.	Czuję się zakłopotany/a, gdy ludzie proszą mnie o powtórzenie	0	1	2	3	4
E29.	Mój głos sprawia, że czuję się niekompetentny/a	0	1	2	3	4
E30.	Wstydzę się moich problemów z głosem	0	1	2	3	4

Adaptacja: B. Miaśkiewicz i wsp., 2022

Światowe Centrum Słuchu
Instytutu Fizjologii i Patologii Słuchu
